# Overview of Current Advances in Extrusion Bioprinting for Skin Applications

**DOI:** 10.3390/ijms21186679

**Published:** 2020-09-12

**Authors:** Arantza Perez-Valle, Cristina Del Amo, Isabel Andia

**Affiliations:** Regenerative Therapies, Biocruces Bizkaia Health Research Institute, Cruces University Hospital, Plaza Cruces 12, 48903 Barakaldo, Spain; arantza.perezvalle@osakidetza.eus (A.P.-V.); cristina.delamomateos@osakidetza.eus (C.D.A.)

**Keywords:** regenerative medicine, skin, bioprinting, extrusion, bioinks, cells

## Abstract

Bioprinting technologies, which have the ability to combine various human cell phenotypes, signaling proteins, extracellular matrix components, and other scaffold-like biomaterials, are currently being exploited for the fabrication of human skin in regenerative medicine. We performed a systematic review to appraise the latest advances in 3D bioprinting for skin applications, describing the main cell phenotypes, signaling proteins, and bioinks used in extrusion platforms. To understand the current limitations of this technology for skin bioprinting, we briefly address the relevant aspects of skin biology. This field is in the early stage of development, and reported research on extrusion bioprinting for skin applications has shown moderate progress. We have identified two major trends. First, the biomimetic approach uses cell-laden natural polymers, including fibrinogen, decellularized extracellular matrix, and collagen. Second, the material engineering line of research, which is focused on the optimization of printable biomaterials that expedite the manufacturing process, mainly involves chemically functionalized polymers and reinforcement strategies through molecular blending and postprinting interventions, i.e., ionic, covalent, or light entanglement, to enhance the mechanical properties of the construct and facilitate layer-by-layer deposition. Skin constructs manufactured using the biomimetic approach have reached a higher level of complexity in biological terms, including up to five different cell phenotypes and mirroring the epidermis, dermis and hypodermis. The confluence of the two perspectives, representing interdisciplinary inputs, is required for further advancement toward the future translation of extrusion bioprinting and to meet the urgent clinical demand for skin equivalents.

## 1. Introduction

Skin is a large and complex organ that serves protective and regulatory functions and is responsible for communication between the external environment and the inner organism. To fulfill these functions, skin has evolved as an organ with a complex anatomy derived from both the ectoderm (epidermis) and mesoderm (dermis). The skin includes not only these two major compartments but also important appendages, including hair follicles, sweat and sebaceous glands, nerve endings, and blood vessels [1], all of which have intricate spatial arrangements that render fabrication of the full skin organ challenging.

Bioprinting technologies, which have the ability to combine various human cell phenotypes, signaling proteins, extracellular matrix (ECM) components, and other scaffold-like biomaterials, are currently being exploited for the fabrication of human skin, broadly aiming to achieve two main goals. The first goal is to meet the urgent clinical demand for skin equivalents, which can range in complexity from advanced dressings for chronic wounds [2] to biomimetic skin grafts to help restore the barrier function in complex ulcers, burns, or traumatic postsurgical wounds [3]. The second important motivation for skin biofabrication is to create disease models for in vitro research and drug development [4,5].

**Figure 1:**
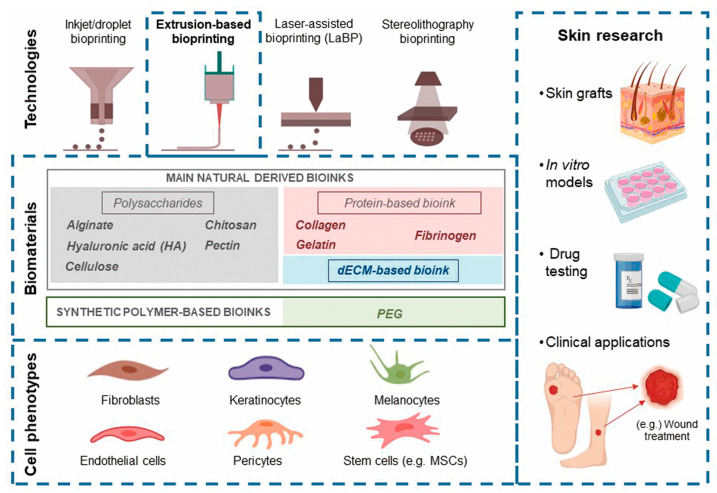
The main bioprinting technologies, printable biomaterials, and cell phenotypes used in skin bioprinting, as well as the main translational applications of this technology.

Among the different bioprinting technologies (i.e., inkjet, laser, extrusion, stereolithography, and microfluidics), extrusion has been identified as the most suitable for manufacturing soft tissue [6] (Figure 1). Thus, we performed a systematic review to estimate the possibilities of extrusion bioprinting for skin applications, describing the main cell phenotypes, signaling proteins, and bioinks (hydrogels) used in extrusion platforms. To understand the current limitations of this technology and how far we are from creating functional skin, we have roughly estimated the maturity of extrusion bioprinting for skin conditions by applying the technology readiness level (TRL) concept to the retrieved studies.

## 2. Skin Biology and Relevant Aspects for Bioprinting

The epidermis, the outermost layer of the skin (0.5–1.5 mm thick), is a thin stratified squamous epithelium that acts as a protective shield for the internal structures of the body, regulates hydration, and provides color to the skin. The complexity of skin originates not only from the myriad of cell phenotypes but also the spatial organization of both the cells and the ECM (i.e., its histology). Therefore, one of the main challenges of skin fabrication using bioprinting techniques is to not only deposit the components of the skin but also to the precisely reproduce a biomimetic tissue. For this, reproducing the architecture is critical; the biomanufactured substitute must present 4 or 5 layers in the epidermis (stratum corneum, stratum lucidum, stratum granulosum, stratum spinosum, stratum basale) and rete ridges. The latter are epithelial extensions that project to the underlying connective tissue (dermis). They are found flattened in scar tissue as well as in most of the biofabricated skin models, thereby reducing epidermal thickness and compromising its barrier function.

High cellularity is a hallmark of the epidermis. Keratinocytes, the most represented cell phenotype, are tightly packed by adherens junctions (cadherins), and produce keratin, which is a resistant and fibrous protein that serves as a barrier. The identification of keratin in the biofabricated skin confirms the epidermal formation, since keratin 10 is a marker of early epidermal differentiation as well as involucrin and filaggrin late differentiation markers [7]. In addition, connected to the keratinocytes and forming the epidermal–melanin unit (EMU), there are melanocytes (melanocyte-to-keratinocyte ratio, 1:36). These cells produce the photoprotective pigment melanin and distribute it to keratinocytes through the EMU, providing color to the skin and protection from UV light. Furthermore, there are less abundant tissue-resident dendritic cells called Langerhans cells, the first immunological defense for the body, and mechanoreceptor cells called Merkel cells, which enable the sensation of touch.

Beneath the epidermis, separated by the basement layer, is the dermis. This is a thick layer of connective tissue composed primarily of two regions, the papillary and reticular dermis, with a low fibroblast density, arranged in a collagenous, anisotropic ECM. In order to assess the dermis growth in biofabricated substitutes, first, the presence of collagen I can be studied, which is an early produced dermis marker; later, fibrillin and elastin are deposited, and hence, they serve as late development markers [8]. Moreover, the presence of collagen IV is considered a mature skin hallmark [3]. The papillary region is tightly connected to the epidermis and provides structural support, cell nourishment, and waste removal. This connection can be pursued by bioprinting manufacturing, and it is commonly assessed by studying the presence of laminin, which is a basement layer protein that participates in the anchoring of the epidermal keratinocytes to the dermis. Blood vessels, nerves, and important appendage structures derived from invaginated epidermis, such as hair follicles and sweat and sebaceous glands, are found in the reticular dermis. Recently, stem cell niches within the skin have been discovered; these cells become multipotent and help in wound healing [9]. The complexity of skin originates not only from the myriad of cell phenotypes but also the spatial organization of both the cells and the ECM (i.e., its histology). Underneath the dermis is the hypodermis or subcutaneous tissue. It remains strongly connected to the dermis, since it contains sweat glands, hair follicle roots, nerves, and large blood and lymphatic vessels en route from the dermis. It is mainly composed of loose connective tissue, e.g., elastin and collagen fibers attached to the dermis, and fat accumulations, helping the skin to maintain the body temperature and acting as a cushion to protect the underlying structures [10].

Owing to its surface location, skin is continuously exposed to external threats, and it is especially sensitive to trauma and disease. Biological insights into the repair process have inspired the design of bioprinting approaches mimicking natural healing mechanisms. When an injury occurs, skin cells sense stressful environmental changes and try to restore skin homeostasis by initiating a dynamic stepwise process, which includes several overlapping biological processes: hemostasis, inflammation, angiogenesis, proliferation, epithelialization, and remodeling. Moreover, this process encompasses complex biochemical changes and crosstalk among multiple cell phenotypes [11]. Thus, the rationale for skin biofabrication should provide for adequate architecture and cellular diversity along with the complex molecular pool essential to fulfill skin functions.

In this context, bioprinting can also benefit from platelet-rich plasma (PRP) biotechnology, as it provides a unique pool of growth factors and cytokines that can enhance healing mechanisms [12,13]. In physiology, upon skin injury and vessel disruption, extravasated blood forms a clot filling the injured area. Activated platelets and leukocytes within this clot release growth factors and cytokines, establishing a cascade of molecular signals that drives tissue repair. Taking advantage of this mechanism, PRP-based therapies have been used to treat nonhealing wounds, with different degrees of success [14,15,16,17]. In fact, the platelet secretome contains more than 300 proteins, and among the crucial effectors of the repair function of PRP are platelet-derived growth factor (PDGF), Transforming growth factor (TGF), fibroblast growth factors (FGF), Epidermal Growth Factor (EGF), Hepatocyte Growth Factor (HGF), Connective tissue growth factor (CTGF), Vascular Endothelial Growth Factor (VEGF) [18]. Accordingly, the inclusion of PRP in bioink formulations can improve the efficacy of biofabricated skin equivalents.

## 3. Overview of Current Research on Extrusion-Based Skin Bioprinting

**Figure 2:**
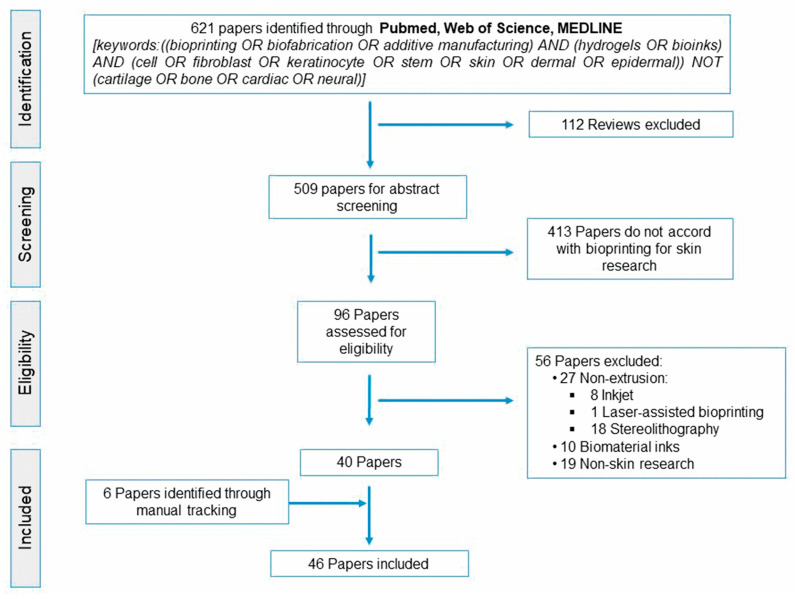
PRISMA flow diagram of the literature search.

Following the PRISMA (Preferred Reporting Items for Systematic Reviews and Meta-Analyses) guidelines, we reviewed research articles published in the last five years to critically appraise advances concerning skin/dermal constructs manufactured through extrusion (Figure 2). We focused on research involving hydrogels (or their precursors) loaded with different cell phenotypes, adhering to the current bioink definition [19]. Thus, we excluded research regarding biomaterial inks not directly formulated with cells or the bioprinting of individual cells.

This field is in the early stage of development, and the bioprinting of personalized, mature skin constructs for implantation is far from being realized; certainly, most of this research is still focused on the improvement of printable biomaterials, mainly in terms of maintaining cell viability while retaining the integrity and resolution of the constructs. The main challenges include controlling the fluid properties of the biomaterial while preserving cell viability during extrusion through small-diameter nozzles and changing the polymer structure by postprinting crosslinking through ionic, covalent, or light entanglement to enable self-support and stability during the deposition of consecutive layers. Moreover, to become specialized bioinks for skin conditions, bioinks must deliver sensitive components, including living cells and signaling factors, in an aqueous environment with adequate pH and osmolarity to favor oxygen and nutrient diffusion, which are paramount for cell survival.

We first addressed printable cell phenotypes in hydrogel scaffolds. Next, we synthesized current research in extrusion bioprinting and classified research studies according to the two main extrusion trends: first, studies involving a biomimetic approach with natural hydrogels, i.e., fibrinogen, decellularized extracellular matrix (dECM), and collagen (Table 1); second, studies focused on polymer bioink modification aiming to enhance the fabrication process (Table 2).

In addition, we roughly estimated the maturity of extrusion bioprinting for skin conditions by applying TRL concepts to the retrieved studies.

### 3.1. Cells Applied in Skin Bioprinting

Skin bioinks (i.e., hydrogels) should mimic the properties of the ECM while preserving cell viability and activities during and after printing. One advantage of extrusion over other bioprinting modalities (i.e., inkjet printing) is that it allows the printing of hydrogels loaded with a high cell density. Commercial cell lines for keratinocytes, melanocytes, hair follicles, and endothelial and dermal fibroblasts are available. Alternatively, specific cell phenotypes can be isolated from skin biopsies. Conventional 2D cultures are commonly used to rapidly generate the millions of cells needed to bioprint tissue. In advanced biofabrication stages, aside from postprinting construct maturation, bioreactors can afford efficient expansion while meeting the tailored requirements for specific cell phenotypes. Various cell phenotypes have been explored to different degrees, including dermal fibroblasts, vascular cells (endothelial, pericytes and microvascular endothelial), keratinocytes, melanocytes, follicle dermal papilla cells, and various sources of stem cells.

The vast majority (61%) of constructs manufactured through extrusion lack complexity and include a single cell phenotype, which is mainly dermal fibroblasts [25,27,29,34,37,38,39,42,45,50,54,57,58,60,61,62]. Although these studies of a single cell phenotype play a role in furthering bioink research, they can only be considered the foundation for creating 3D-bioprinted skin constructs. Even though these methods can be applied to manufacture dermal constructs in an automated way, the resulting constructs differ little from hand-poured hydrogels seeded with fibroblasts. Unfortunately, these models fail to represent the entirety of the functions of the skin, which requires more complex systems integrating multiple cell phenotypes with complex molecular crosstalk.

Fibroblasts are crucial for dermal formation and wound repair, as in the presence of appropriate stimuli, including but not limited to PDGF, IGF-I, and TGF-β1, they synthesize ECM-forming proteins and additional signaling factors. The latter are involved in both autocrine (i.e., TGF-b, connective tissue growth factor (CTGF), VEGF, PDGF-BB) and paracrine (i.e., ICAM-1, VCAM-1, IL-6, IL-8, IL-15, MMPs, CCL2, CCL7, TIMP-1) signaling; thus, they not only participate in fibroblast communication but also coordinate their activities with surrounding cells, i.e., immune cells, endothelial cells, and stem cells in niches [12,64].

However, poor advances in bioprinting blood and lymphatic vessels have limited the translational application of skin constructs. The vascular and lymphatic systems located in the dermis are essential for the proper distribution of oxygen and nutrients and removal of waste, respectively. In addition, they are involved in inflammatory skin conditions and wound healing. Despite their importance, only 7 of the 47 articles reviewed reported the blending of fibroblasts with endothelial cells or pericytes [3,4,7,20,26,30,43].

The primary function of the skin, i.e., serving as a barrier to pathogen invasion, requires a healthy epidermal layer made mainly of keratinocytes. Altered barrier function is involved in inflammatory skin conditions [4]. However, only two works employed melanocytes [20,31], which fabricate the photoprotective pigment melanin. The interplay between the two main cell phenotypes of the epidermis, i.e., keratinocytes and melanocytes, is crucial to form the EMU and distribute melanin to keratinocytes, supporting the protective function of the skin against light and heat.

Moreover, the interaction between fibroblasts and keratinocytes is required for the recovery of skin homeostasis. Indeed, keratinocytes instruct fibroblasts to produce several tissue-forming factors, i.e., keratinocyte growth factor (KGF), fibroblast growth factor (FGF), IL-6, GM-CSF, hepatocyte growth factor (HGF), IL-6, IL-19, and PDGF-BB [64,65]. Nonetheless, merely eleven of the reviewed articles introduced fibroblasts and keratinocytes together in their models [22,23,24,31,32,66], and more importantly, only five of these works have successfully created vascularized, full-thickness skin substitutes [3,4,7,20,26].

### 3.2. Stem Cell Sources

Despite the potential envisioned for mesenchymal stem cells (MSCs) and their secretome in tissue engineering [67], interest in combining bioprinting and stem cell research has grown recently. Different stem cell sources were used in 15 of the works, including bone marrow, adipose tissue, perinatal tissues (umbilical cord, Wharton’s jelly), and amniotic fluid.

The most commonly used stem cells are bone marrow-derived mesenchymal stem cells (BM-MSCs), as they were the first to be isolated [28,43,46,47,55,56,63]. They have a multilineage differentiation capacity and can differentiate into several cell types, including skin-like cells, i.e., fibroblasts [46], keratinocytes, endothelial cells, and pericytes [68]. Moreover, during physiological wound healing, circulating MSCs are recruited to the wound site and differentiate into skin cell phenotypes [68].

Adipose tissue-derived stem cells (ASCs) are a more advantageous type of MSC. Unlike BM-MSCs, ASCs can be easily isolated in large quantities from abundantly available human adipose tissue through a minimally invasive procedure. ASCs have also shown potential in wound healing. They can differentiate into keratinocytes, fibroblasts, and endothelial cells, as well as release a healing milieu of cytokines and growth factors that support angiogenesis, fibroblast migration, and fibronectin and collagen production [69]. Therefore, their use is considered promising in skin regeneration, but as they were discovered later, only five of the reviewed studies used ASCs [7,26,36,40,55], and these studies mainly assessed cell viability. Only Kim BS et al. [7,26] proved the in vivo wound-healing properties of the fabricated scaffolds, reinforcing the benefits of including stem cells in bioprinted grafts.

On the other hand, pluripotent stem cells have further advantages, as they can differentiate into any somatic cell type of the body. Among these, embryonic stem cells (ESCs) are derived from the inner cell mass of blastocysts [48,51], and induced pluripotent stem cells (iPSCs) [4,33,59] are derived from somatic cells that have been reprogrammed to induce pluripotency. However, safety concerns linger because of their teratogenic potential. In addition, in the case of ESCs, very few cells are obtained from each extraction, and there are ethical concerns due to their embryonic origin. Therefore, there are critical issues regarding the application of these cells for clinical purposes, and their implementation in human therapy is challenging.

Very recently, human amniotic epithelial cells (AECs) have emerged as a safer source of pluripotent stem cells. They can be easily isolated from the inner amniotic membrane of the placenta, without invasive procedures or associated ethical issues. AECs have shown promising results in wound healing [70,71] and become great candidates for skin tissue engineering. In this way, Liu P et al. [41] developed a scaffold containing AECs and a special type of MSCs derived from the umbilical cord, Wharton’s jelly-derived MSCs (WJMSCs). While AECs are more likely to differentiate into keratinocytes, WJMSCs differentiate into fibroblasts and endothelial cells. Thus, in this work, they explored the development of a meaningful multi-layered skin construct with epidermal (AECs) and dermal (WJMSCs) compartments.

Despite the extensive number of cell phenotypes used in the reviewed studies, no single work has employed lymphatic, nerve, or sweat gland cells. These are strikingly important phenotypes for the recovery of skin physiology and homeostasis after injury, so research in this field needs to keep evolving to precisely mimic human skin and ensure clinical applications.

## 4. Cell-Laden Bioinks

Our systematic review confirms the two major trends in bioprinting research [6]. On the one hand, some studies focused on the biomimicry strategy, aiming to replicate cellular and extracellular structures with better strategies for maturation and remodeling (Table 1). On the other hand, we identified a large number of articles whose main focus was leveraging advanced biomaterials to ease the extrusion process while achieving constructs with good mechanical stability and preserving cell viability (Table 2).

### 4.1. Fibrinogen-Based Bioinks

Fibrinogen is an abundant plasma protein that is fluidic while circulating in the bloodstream but turns into a natural hydrogel-like matrix by the action of thrombin. The resulting fibrin matrix is stabilized by coagulation factor XII and mimics the provisional ECM in early healing. Moreover, when used with platelets in the form of PRP, it provides a physiological milieu of growth factors and cytokines that influence cell activities and support wound healing [18]. Indeed, PRP supplies cells with the appropriate stimuli for the proliferation, migration, and ECM protein synthesis. PRP or merely fibrinogen has been used in bioink formulations as a single-component bioink [24] or mixed with other molecules, including but not limited to collagen [21,22,66], dECM [7], alginate [30], gelatin [20,23,43], and others [4].

Taking advantage of the hydrogel-forming capacity of fibrinogen and signaling factors of the platelet secretome [72], Cubo N. et al. [24] created a dermal compartment composed of fibroblasts and human plasma containing fibrinogen. Additionally, they manually deposited an epidermal layer of keratinocytes, fabricating a bilayered skin substitute that showed structural and functional similarities to human skin when grafted on mice. Progress in automation was achieved by developing a multisyringe extrusion system, allowing the simultaneous extrusion of bioinks, crosslinkers, and stabilizers [73]. Moreover, a particular biomimetic approach using fibrinogen blended with gelatin and hyaluronic acid (HA) enabled the manufacturing of facial skin grafts customized using medical images [23]. Additional complexity was achieved by bioprinting skin equivalents, comprising not only dermis and epidermis but also the basal layer [41].

Recently, Jorgensen et al. [20] reported the automated fabrication of full-thickness skin equivalents combining six different human cell phenotypes using a bioink composed of fibrinogen, gelatin, and HA, and a three-extruder bioprinter. The three-layer constructs included most of the cell types present in native human skin—namely, keratinocytes and melanocytes for the epidermis; fibroblasts, microvascular endothelial cells, and follicle dermal papilla cells for the dermis; and preadipocytes for the hypodermis. Although these grafts favored acute wound closure overall, in an athymic mouse model, there were no differences in individual healing parameters, such as wound contraction and epithelialization at 21 days. Despite these limitations, this work represented a proof of concept of the potential of fibrinogen-based bioinks for manufacturing skin constructs, which were successfully remodeled in vivo.

### 4.2. dECM-Based Bioinks

Providing a natural microenvironment to cells and tissues is one of the most effective ways to ensure the success of regenerative therapies. 3D bioprinting allows the use of dECM-based bioinks, overcoming the challenge of specifically mimicking the natural microenvironment of a given tissue. For this, the cellular component of a concrete ECM is removed following different chemical (acids/bases, detergents, hypotonic/hypertonic solutions, alcohols), physical (temperature, pressure, electroporation, force), and/or biological (enzymes, chelating agents) processes, maintaining its structure, basic functionality, and components, i.e., growth and differentiation factors specific to the target tissue. Moreover, after the removal of local cells, the inflammatory response or immune rejection is avoided [25,74].

Ahn G. et al. [27] examined the printability of different concentrations of porcine-derived skin dECM (s-dECM) bioinks by analyzing the trade-off between fluidity, gelation, shape retention, and ability to preserve the viability of embedded mouse fibroblasts during and after printing, but they overlooked cell activities. Likewise, Won J.Y. et al. [25] formulated a bioink containing porcine dermis dECM and human dermal fibroblasts (HDFs) and confirmed the suitability of dECM over collagen bioinks. In fact, the embedded fibroblasts showed over 90% viability, and their bioink promoted fibroblast proliferation, together with the enhanced expression of genes implicated in skin morphology and development.

Kim B.S. et al. [26] went one step further and proposed a full-thickness skin substitute blending s-dECM and HDFs. This bioink supported cell viability and proliferation, keratinocyte adhesion, and proper stem cell differentiation compared to collagen bioinks in vitro. To construct the epidermal layer, human epidermal keratinocytes (HEKs) resuspended in culture medium were deposited using inkjet bioprinting. The functionality of the newly formed tissue was greater than that of tissue developed from collagen bioink, as the anisotropy and barrier function of the former were more similar to those of native skin.

Furthermore, prevascularized skin patches created with endothelial progenitor cell (EPC)- and human adipose-derived stem cell (hASC)-laden s-dECM bioink were implanted in BALB-c mice for proof of concept. They performed better than collagen I hydrogels as assessed by wound closure, neovascularization, and re-epithelialization.

More recently, Kim B.S. et al. [7] leveraged their skin construct by developing a new model of full-thickness, vascularized skin based on dECM and fibrinogen bioinks, which provided a microenvironment closer to that of native skin than previously described, in addition to providing cell-instructing factors [7]. First, they bioprinted the hypodermis bioink, which was composed of adipose-derived dECM and fibrinogen, with preadipocytes blended within. On top, they deposited a vascularized scaffold by casting a cylindrical tube across the structure made of gelatin, thrombin, and human umbilical vein endothelial cells (HUVECs). Then, the dermal compartment, containing skin-derived dECM, fibrinogen, and HDFs, was extruded on top of the previous layers. During printing, thrombin was sprayed onto the hypodermal and dermal compartments, and immediately after fabrication of the scaffold, it was thermally crosslinked at 30 °C and 37 °C. After 7 days of maturation in vitro, the outermost epidermal layer containing HEKs was deposited to complete the functional, full-thickness skin substitute. In this way, these researchers overcame translational hurdles, ensuring a construct for the in vitro modeling of skin-related pathologies, such as diabetic foot ulcers and psoriasis.

### 4.3. Collagen-Based Bioinks

Following a biomimetic approach, collagen type I has been proposed as the main component in bioink in several studies [3,28,29,30,31,52]. Collagen type I can reproduce a natural environment with Arg–Gly–Asp (RGD) domains and replicate skin porosity and anisotropy. Indeed, collagen type I is found in all dermal layers, being the main component of the reticular dermis and the most commonly used natural polymer in tissue engineering. However, it has a slow gelation rate and requires careful selection of the buffer composition, pH, and extruder temperature to avoid clogging, as well as efficient postprinting crosslinking to retain integrity. Although pure collagen is successfully used in other novel 3D cell culture technologies, such as microfluidics [75,76], to meet the printability requirements, methods for rapid crosslinking while maintaining high cell viability need to be developed [3]. The chemical reticulation of collagen through methacrylation [28] or the addition of photoinitiators [31] can improve hydrogel stability.

A commercial collagen-based bioink named Viscoll^TM^, which can be complemented with ECM proteins and growth factors, was developed by adjusting the kinetics of polymerization through precise control of the temperature in the bioink/bioprinter platform [29]. In fact, the formulation of hybrid bioinks leveraged the printability and cytocompatibility of collagen [52]. By depositing collagen, alginate, and fibrin through a multiaxial extrusion system, Attalla R. et al. [30] created tubular, bilayered, and trilayered structures that allowed cell proliferation and adhesion. To avoid the use of alginate and its poor biodegradability, collagen was supplemented with tyrosinase and methacrylate gelatin (GelMA) [31]. Tyrosinase facilitated GelMA-collagen crosslinking, improving the mechanical properties of the hydrogel, and proof-of-concept in vivo animal studies showed enhanced epidermal and dermal regeneration.

In summary, with collagen, printable, stable constructs can be achieved by photochemical reticulation through careful temperature control and using hybrid bioinks created through the addition of other biomaterials.

### 4.4. Alginate-Based Bioinks

The most investigated bioinks are based on alginate (Table 2). Alginate is a natural ionic biocompatible polysaccharide that is highly hygroscopic and commonly used as a dressing for exudative wounds [77]. The properties of alginate, i.e., flexibility, gelation time, and pore size, can be tuned to meet the bioprinting requirements by controlling the relative ratio between the two block components (mannuronic acid and glucuronic acid) and chiefly crosslinking glucuronic with divalent ions (Ca^2+^ Sr^2+^, Ba^2+^). Research efforts have been focused on reducing the alginate concentration in bioink, thus increasing the pore size and gas and nutrient exchange, thereby enhancing cell viability and proliferation [37,78,79].

However, low concentrations also decrease the bioink viscosity and thus affect the shape fidelity and construct stability. Polymer networks have been strengthened by chemical modification with peptide domains (P1) and combination with a recombinant engineered protein (C7) [38]. The modified alginate formed an extrudable soft hydrogel that preserved the hydration and viability of hASCs and fibroblasts during manufacturing. The mixture of alginate and cellulose derivatives has become a successful strategy to reduce the weaknesses of low concentrations of alginate, as has been proven by not only the blend of alginate (3% *w*/*v*), methylcellulose (9% *w*/*v*), and trisodium citrate [57] but also the blend of alginate (3% *w*/*v*), carboxymethylcellulose (3% *w*/*v*), and cellulose nanofibrils (1.5% *w*/*v*) [54]. These bioinks exhibited good printability, stability, and shape fidelity, as well as excellent viability. In addition, efficient alginate network strengthening was also achieved with norbornene (cyclic alkene), which enabled ultrafast, strong, light-triggered crosslinking via a photoinitiated thiol-ene reaction [35]. In this way, the alginate concentration could be reduced while RDG domains, facilitating cell adhesion, were introduced by means of a thiol-adhesive peptide (HS-RGD). Thus, complex geometries could be bioprinted while maintaining cell viability above 80% [35].

The functionalization of alginate by adding different nanocomposites and enantiomers, i.e., PMOs-(L)-Asp-alginate and PMOs-(D)-Asp-alginate, favored fibroblast accumulation and improved cell activities [34]. Moreover, cell adhesion and migration were dependent on the chirality of the added molecules, as fibroblasts accumulated preferably in the hydrogel containing the D-aspartic acid enantiomer. This strategy opens the door to the generation of advanced systems providing the spatial positioning of cells.

In summary, alginate is widely used for its biocompatibility and cytocompatibility. However, additional strategies are required to overcome the two major drawbacks. First, its poor shape fidelity at low concentrations highlights the need for crosslinking strategies to achieve shape retention and suitable porosity. Second, as it lacks RGD domains, which enable cell adhesion, natural proteins, including fibrin, chitosan, collagen, HA and most commonly gelatin, are commonly blended with alginate [44,80] to provide a better microenvironment for cell activities.

### 4.5. Gelatin-Based Bioinks

Gelatin is a high-molecular-weight polypeptide often obtained from the hydrolysis of collagen, which is extracted from connective tissues, i.e., bones, tendons, or skin. As a nontoxic and biocompatible material, it has been commonly used in various biomedical applications. Biodegradability, low antigenicity, and the presence of RGD domains make it an optimal hydrogel for cell adhesion, growth, and proliferation [81]; thus, it is suitable as a bioink component.

To improve the mechanical properties of gelatin, the polymer backbone can be chemically modified through derivatization with methacrylic anhydride. Reticulated gelatin, i.e., GelMA, maintains the biological properties intrinsic to gelatin, such as RGD domains, while enabling covalent crosslinking [82]. Moreover, the introduction of photoinitiators, such as 2-hydroxy-4′-(2-hydroxyethoxy)-2-methylpropiophenone (Irgacure D-2959) or lithium phenyl-2,4,6-trimethylbenzoylphosphinate (LAP), facilitates the formation of stronger constructs through tunable dual crosslinking mechanisms [39]. Optimization of the GelMA concentration, the extent of functionalization, and/or the UV intensity can improve the stiffness and pore size of the construct and thus cell spreading [40]. Other limitations of GelMA, such as photocuring kinetics, filament spreading, or cell viability, have been improved by its modification with norbornene moieties (GelNB) [39].

Additional reinforcement techniques, such as supplementation with alginate derivatives (e.g., AlgHEMA) and silicate nanoparticles (SiNPs), have also improved printability and long-term stability without compromising cell spreading or proliferation [40]. Temperature control, e.g., warming cell-laden prebioink (GelMA) at 37 °C followed by postprinting cooling to 4 °C, favored the fabrication of stable constructs [41]. Alternatively, adding transglutaminase enzyme (TG) can improve viscosity by forming covalent bonds [45]. In addition, thermoplastic reinforcement with polyethylene glycol (PEG) can favor gelatin stiffness [43].

Furthermore, mixing gelatin with alginate can enhance extrudability through component rate and temperature control [47,51] (both polymers are thermoresponsive) and facilitate postprinting crosslinking [46,47,50].

### 4.6. Other Hybrid Bioinks

The greatest challenge to be addressed in the development of composite bioinks is twofold: to make a biologically relevant material extrudable while keeping its shape postprinting. For example, cellulose-derived materials, which provide viscosity [83], can be doped with growth factors and cytokines using platelet lysates [55] or enhanced with HA [56].

Novel approaches to mix components include a double-extrusion platform capable of the layer-by-layer deposition of the antibacterial and antifungal polysaccharide chitosan and poly(gamma-glutamic acid) (gamma-PGA) [58]. The formation of electrostatic interactions between the amino groups of chitosan and the carboxylic groups of gamma-PGA provided the printed construct with stability while ensuring good cell viability.

Furthermore, nonionic thermosensitive crosslinking, i.e., transforming chitosan into hydroxypropyl chitin (HPCH), favored hiPSC survival and proliferation along with pluripotency maintenance [59]. Similarly, the addition of hydrazone to HA [60] promoted cell migration [84,85], proliferation, and motility, angiogenesis [86], and wound healing [87].

However, chemical modifications can be detrimental for cell viability. Therefore, an alternative methodology based on inorganic sol–gel polymerization, i.e., controlling the reticulation of hydroxypropyl methylcellulose (HPMC) by tuning its silylation ratio, has been explored [53].

## 5. Skin Bioprinting, from Bench to Society, Technology Readiness Pathway

Despite advances in cell-laden biomaterial printability, extrusion bioprinting is far from being able to produce functional skin to meet the existing clinical demand.

We have used technology readiness levels (TRLs), comprising nine levels, as a valid metric to assess the evolution and readiness of bioprinting from fundamental research to competitive manufacturing (Table 3). TRLs help to identify research priorities to delineate the pathway from experimental research to applications in society. Assessing TRLs also serves to determine the corresponding manufacturing readiness levels (MRLs) used to identify risks and gaps from the manufacturing perspective [88].

**Figure 3:**
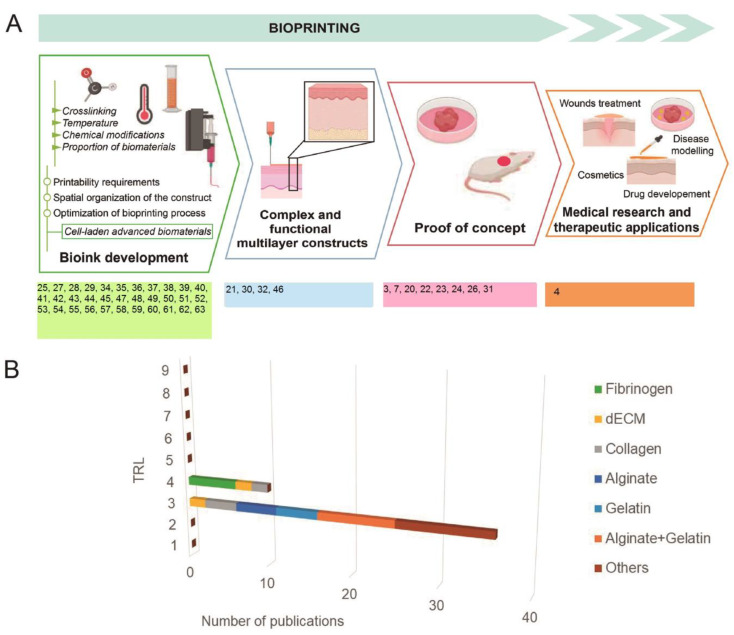
(**A**) Display of the reviewed publications according to the stage of development; (**B**) Distribution of analyzed bioinks for extrusion bioprinting according to technological development.

As depicted in Figure 3, most of the reviewed works focus on the first steps of the development. In fact, proof of concept was only achieved in eight of the works, which were the biomimetic bioinks from studies shown in Table 1. This demonstrates the immaturity of the technology, which is seldom validated in experimental research in vivo; no developments have reached sufficient maturity to be applied in a clinically relevant environment.

While several companies focused on the bioprinter market business have reached TRL9 [91,92], the biofabrication of tissue equivalents for skin conditions is still in the early phase of laboratory research. In particular, research studies dealing with extrusion bioinks are at TRL3, while a few studies have progressed to TRL4 with testing of the bioprinted product in animal models (Figure 3). Indeed, no publications/clinical trials concerning the use of bioprinting for skin conditions in humans have been reported.

## 6. Limitations and Future Directions

Extrusion bioprinting is highly interdisciplinary, as it involves the development of complex platforms requiring interdisciplinary knowledge, including knowledge of medical imaging, hardware, and software to control multiple extruders (the printer), and advanced biomaterial development together with cell production, including a deep understanding of physiology and cellular biology. Moderate progress in extrusion bioprinting has led to a novel technology, which involves bioink extrusion in a yield stress fluid that is capable of supporting the extruded bioink (reviewed in [6]), joining competing requirements from the perspective of manufacturability (engineering) and biomimetics (life sciences).

Further advances in tissue engineering and regenerative medicine are expected from the convergence of cell electrospinning (i.e., using a coaxial bio-electrospray) with 3D bioprinting. The former allows the controlled distribution of cells, encapsulated in nanosized fibers (reviewed in [92]). In addition, commonly bioprinted constructs are not ready for in vivo applications and have to follow a maturation process, where architectural changes and remodeling are recognized as the fourth dimension of bioprinting [93]. Alternatively, remodeling can take place in the host tissue. In this context, the merger of robotics with bioprinting has evolved toward intraoperative bioprinting, spanning from engineering, cellular biology, and biomaterials to medical sciences and surgery [94]. However, such far-reaching frontiers have inflated expectations because of the promising benefits achieved thus far.

Many intricate challenges need to be overcome before bioprinting technology achieves its full potential and transcends the accomplishments of tissue engineering. First, the so-called bioinks, i.e., cell-laden advanced biomaterials or natural polymers, have to be optimized to meet the requirements for printability, reproducibility, and spatial organization of the construct; second, the living skin equivalent should be doped with a molecular pool of signaling proteins for the activation of healing mechanisms in a manner that can address the specific requirements of the skin as an organ and various medical conditions. The inclusion of cell signaling molecules in bioinks is often neglected, broadening the disparity between the in vitro and in vivo microenvironments. Thus, the confluence of the two perspectives, representing interdisciplinary inputs as reflected in bioink development, i.e., biomimicry and manufacturability, are required for further advancement toward the future translation of biofabrication.

Based on the original works identified in this review, technology transition to commercial products could be anticipated in the near future in the field of wound management. In order to meet the market and clinical demand, these bioprinted constructs should enable tissue repair and the reconstruction of skin architecture in clinically relevant contexts, such as diabetic or vascular ulcers or burn wounds. To shorten the time to market, experimental research (TRL4-5) should generate data that are ready to be used in the certification of the bioprinted construct. A co-development interdisciplinary methodology should achieve constructs with high performance–cost ratio and, generate clinical data that meet regulatory issues associated with marketing authorization of the living constructs (i.e., Advanced Therapy Medicinal Products, ATMP).

Although we are still far from skin fabrication for regenerative medicine, the applications of bioprinted constructs also expand to the generation of in vitro models for drug discovery, which is technically easier with less regulatory constraints. These features help to speed TRL development and get earlier the market demand, while leveraging our accomplishments in biofabrication.

## Figures and Tables

**Table 1:** Studies involving a biomimetic approach with natural hydrogels (fibrinogen, dECM and collagen).

**Fibrinogen**						
**Author Reference**	**Biomaterial**	**Cell Phenotypes (Source, Density (cell/mL))**	**Rheology**	**Bioprinting Conditions**	**Post-Printing Processing**	**Bioprinted Construct Application/Evaluation**
Jorgensen A.M. 2020 [20]	Fibrinogen (30 mg/mL), Glycerol (100 µL/mL), Gelatin (35 mg/mL), HA (3 mg/mL), Aprotinin (40 µg/mL)	*Epidermis:* (ratio 9:1) hKCs, hMCs. *Dermis:* hFBs, FDPCs, hDMECs. *Hypodermis:* pre-adipocytes. Total cell concentration: 20 × 10^6^	NO	Extrusion: Pneumatic; N. extrusors: Three; Nozzle Ø: 500 μm metal; Pressure: 60~90 kPa	Thrombin (20 IU/mL, 60 min, RT)	Proof-of-concept validation of full-thickness bioprinted skin constructs for wound closure. Testing and evaluation of printed skin grafts in mice. Construct evaluation: -SEM: analysis of the structure and morphology of the construct. Histology: H&E, Masson’s trichrome, and picrosirius red. Immunostaining: Lamin A + C, Pan-cytokeratin, Mel5, CD146, adiponectin, vimentin, ZO-1, keratin71
Liu X. 2020 [4]	Fibrinogen (2.5 mg/mL), NovoGel component 2 (60 mg/mL)	*Epidermis:* hNHKs (2 × 10^5^ cell/cm^2^)—manually seeded; *Dermis:* hNFBs (8 × 10^6^), hiPSC derived endothelial cells (7 × 10^6^), placental microvascular hPCs (0.7 × 10^6^)	NO	Nozzle Ø: 250 µm	Thrombin (1 U/mL, 24 h)	Bioprinting of vascularized full-thickness skin tissue equivalent of atopic dermatitis model for preclinical studies. Construct evaluation: Trans-epidermal electrical resistance measurement. Histology: H&E. Immunostaining: human collagen IV, laminin 5, integrin β, filaggrin, KRT10, loricrin, E-cadherin, CD-31, phalloidin, desmoglein, claudin-1. Cytokine measurement: ICAM, VCAM, VEGF-A, VEGF-C, VEGF-D
Derr K. 2018 [21]	*Basal layer:* Laminin/Entactin (1.61 mg/mL in DMEM)*; Dermis:* Fibrinogen (7.7 mg/mL), Gelatin (0.045 mg/mL), Collagen I (4 mg/mL), Elastin (0.55% *v*/*v*)	*Epidermis:* hNKCs (6.15 × 10^6^); *Dermis:* hNFBs (2 × 10^6^)	NO	*Epidermis:* Extrusion: pneumatic; *Basal layer:* Extrusion: jetting; *Dermis:* Extrusion: plunger; N. Extrusors: three	Thrombin (5 U/mL, 1.5 h, RT)	Fabrication of morphologically and physiologically relevant skin substitutes. Construct evaluation: Histology: H&E. Immunostaining: collagen I, collagen VII, Ki67, cytokeratin 15, ZO-1, claudin 1, e-cadherin, phalloidin, filaggrin. OCT imaging. Permeability. Barrier function
Hakimi N. 2018 [22]	*Epidermis:* Fibrinogen (2.5%), HA (0.25%); *Dermis:* Fibrinogen (1.25%), HA (0.25%), Collagen I (2.5 mg/mL), Alginate (1%)	*Epidermis:* hKCs (1.5 × 10^6^)*; Dermis:* hFBs (4 × 10^5^)	YES	Speed: 0.3–1.6 cm^2^/s	Thermal gelation, 30 min, CaCl_2_ (10 mM), Thrombin (50 IU)	Development of handheld printer for in situ bioprinting. Proof-of-concept in mice and porcine wound model. Construct evaluation: SEM for surface microstructure. Histology: H&E. Immunostaining: phalloidin, F-actin, keratin 14, keratin 10, α-SMA
Seol Y.J. 2018 [23]	Fibrinogen (20 mg/mL), Gelatin (30 mg/mL), HA (3 mg/mL), Glycerol (10% *v*/*v*)	*Epidermis:* hKCs (1 × 10^7^); *Dermis:* hFBs (5 × 10^6^)	NO	Extrusion: Pneumatic; Nozzle Ø: Teflon 300 µm; Pressure: 60 kPa	Thrombin (20 U/mL)	Bioengineered skin substitute combined with a wound dressing layer for facial wounds; Construct evaluation: Wound contraction measure in vivo. Histology: H&E
Cubo N. 2017 [24]	*Dermis:* Fibrinogen (2.3 mg/mL); Tranexamic acid, CaCl_2_ (0.1%)	*Epidermis:* hKCs (6 × 10^6^); *Dermis:* hFBs (1.75 × 10^4^)	NO	Extruders: two; Flow: 12 mL/min	*Dermis:* 37 °C, 30 min	Functional human bi-layered skin tested in immunodeficient mice model; Construct evaluation: Histology: H&E. Immunostaining: vimentin, keratin 5, keratin 10, filagrin, collagen VII, SMA
**Decellularized Extracellular Matrix (dECM)**						
**Reference**	**Biomaterial**	**Cell Phenotypes (Source, Density (cell/mL))**	**Rheology**	**Bioprinting Conditions**	**Post-Printing Processing**	**Bioprinted Construct Application/Evaluation**
Kim B.S. 2019 [7]	*Dermis*: s-dECM (1.5%), Fibrinogen (10 mg/mL), NaCl (1.1%), Aprotinin (5 µg/mL); *Vasculature*: Gelatin (10%), Glycerol (10%), Thrombin (100 U/mL); *Hypodermis*: a-dECM (2%), Fibrinogen (10 mg/mL), NaCl (1.1%), Aprotinin (5 µg/mL)	*Epidermis:* hKCs (5 × 10^6^); *Dermis:* hFBs (5 × 10^5^); *Vasculature:* HUVECs (1 × 10^7^); *Hypodermis:* Pre-adipocytes (1 × 10^6^)	NO	*Hypodermis, Dermis* and *Vasculature:* Extrusion; *Epidermis:* Inkjet	*Dermis and hypodermis:* (I) Sprayed thrombin (100 U/mL), (II) 30 °C, 10 min, III) 37 °C, 30 min	Development of a novel printing platform for a full-thickness skin model using dECM with a vascular channel. Construct evaluation: Histology: H&E, Masson’s trichrome., Immunostaining: CD31, keratin 10, filaggrin, laminin, collagen type I, fibronectin, BODIPY, p63, keratin 19, Ki67. Permeability of vascular channel
Won J.Y. 2019 [25]	dECM (2–3%)	hFBs (1.5 × 10^6^ cells)	YES	Nozzle Ø: 500 µm	37 °C, 30 min	Promotion of skin regeneration as well as the survival and proliferation of skin-derived cells by the application of dECM cell-laden bioink to form skin substitutes. Construct evaluation: Microarrays for gene expression of ECM, skin development and morphology
Kim B.S. 2018 [26]	*Dermis* and *Hypodermis:* Porcine s-dECM, Acetic acid (0.5 M), Pepsin	*Epidermis:* hKCs (6 × 10^6^); *Dermis:* hFBs (5 × 10^5^), EPCs (2.5 × 10^6^); *Hypodermis:* hASCs (2.5 × 10^6^)	YES	*Epidermis:* Inkjet; Nozzle Ø: *Epidermis:* 120 µm, *Dermis* and *Hypodermis:* 600 µm	37 °C, 30 min	Fabrication of human full-skin pre-vascularized equivalent using dECM by printing different layers. Construct evaluation: Transepithelial electrical resistance, -Water permeability of the construct. SEM for bioink microstructure. Histology: H&E, Masson’s trichrome, Alcian blue. Immunostaining: keratin 10, involucrin, collagen type-I, fibronectin, decorin, laminin. Gene expression: collagen type-I, fibronectin, decorin, collagen type-III, vimentin, keratinocyte growth factor. In vivo wound healing. In vivo construct histology: H&E, re-epithelialization. In vivo construct immunostaining: CD31, cytokeratin. In vivo blood flow measurement
Ahn G. 2017 [27]	s-dECM (2.5%) Acidic pepsin	mFBs	YES	Extrusion: pneumatic; Nozzle Ø: 250 µm, Pressure: 60 kPa, Speed: 125 mm/min	*During printing:* Heating the nozzle and bed at 37 °C	Development of printing strategy of cell-laden dECM constructs by inducing simultaneous gelation. Construct evaluation: SEM to measure pore size. Immunostaining: F-actin
**Collagen**						
**Reference**	**Biomaterial**	**Cell Phenotypes (Source, Density (cell/mL))**	**Rheology**	**Bioprinting Conditions**	**Post-Printing Processing**	**Bioprinted Construct Application/Evaluation**
Baltazar T. 2020 [3]	*Epidermis:* KGM, Skin differentiation medium; *Dermis*: Collagen I (3.5 mg/mL), FBS (5%), pH reconstruction buffer (1X- 290 µL), HAM-F12 medium (290 µL)	*Epidermis:* hKCs (1 × 10^6^)*; Dermis:* hFBs (7 × 10^5^), hECs (7 × 10^5^), hPCs (3.5 × 10^5^)	NO	Extrusion: Pneumatic; *Epidermis:* Nozzle Ø: 100 µm; Pressure: 35 kPa for 54 s; *Dermis:* Nozzle Ø: 150 µm, Temp: 4 ºC, Pressure: 50 kPa for 205 s	37 °C	Fabrication of 3D bioprinted bilayered skin grafts. Construct evaluation: Histology: H&E. Immunostaining: filaggrin, cytokeratin 14, cytokeratin 10, collagen type-IV, Ki67, laminin 5, CD31. Endothelial network stability. In vivo graft histology: H&E, vascularization. In vivo graft immunostaining: cytokeratin 14, cytokeratin 10, Lectin I, GSL-B_4_, laminin 5, CD31, F4/80, involucrin. In vivo vascularization by perfusion
Kajave N.S. 2020 [28]	CMA (3 mg/mL), VA-086 photoinitiator (1%)	hMSCs (1 × 10^5^)	YES	Nozzle Ø: 210 µm, Flow: 5 mm/s	(I) UV (365 nm/17 mW/cm^2^, 1 min); (II) Genipin (0.5 mM or 1 mM, 1 h, 37 °C)	Development of stable and printable CMA hydrogels with dual crosslinking process
Osidak E.O. 2019 [29]	Collagen Viscoll^TM^ (collagen I, 20, 30, and 40 mg/mL) neutralized in acetic acid (20 mM)	mFBs (0.5 × 10^6^)	YES	Nozzle Ø: 250 µm, Temp: 15 °C, Flow: 5 mm/min	Printing bed at 37 °C for instant gelification	Adaptation of commercial Viscoll collagen to a bioink for 3D bioprinting of cell-laden constructs
Attalla R. 2018 [30]	CaCl_2_ (100 mM); Alginate (0.5%), Collagen (2.5 mg/mL) or Alginate (1%), Fibrinogen (25 mg/mL)	HUVEC + RFP; mFBs + GFP; Cell concentration: 2 × 10^6^	YES	N. extrusors: three. (I) Nozzle Ø: 260 µm; (II) Nozzle Ø: 630 µm; (III) Nozzle Ø: 830 µm. Flow: 1–6 mL/min; Speed: 1–16 m/min	*Fibrinogen bioink:* Thrombin (250 U/mL, 30 min)	Fabrication of complex heterogeneous bi- and tri-layered hollow channels within multi-layered scaffolds using multi-axial nozzle. Construct evaluation: Cell distribution in the hollow channels
Shi Y. 2018 [31]	Collagen I-rat (8%), GelMA (5%), Tyrosinase (300 U/mL), I2959 photoinitiator (0.1%)	hMCs (3 × 10^4^), hKCs (1 × 10^6^), hFBs (1 × 10^6^)	YES	Nozzle Ø: 200 µm, Temp: 17 °C, Pressure: 0.8–1.2 bar, Speed: 7–10 mm/s	UV (365 nm, 40 s)	Development of skin substitutes with GelMA bioink doped with tyrosinase enhancing the wound closure in vivo (rats) and prevention of scar formation. Construct evaluation: SEM: cell morphology. Histology: H&E. Wound closure measurement
Kim B.S. 2017 [32]	*Dermis:* Collagen I-porcine skin (2%)	*Epidermis:* hKCs (1 × 10^6^)*; Dermis:* hFBs (2 × 10^5^)	NO	*Epidermis:* Inkjet; *Dermis:* Extrusion: pneumatic. Pressure: 5–200 kPa	37 °C, for at least 30 min	Development of a hybrid and versatile 3D direct cell-printing system for human skin model biofabrication. Construct evaluation: Histology: H&E. Immunostaining: collagen type I, keratin 10, involucrin. Epidermis thickness

a-dECM, adipose-derived decellularized extracellular matrix; CMA, acid solubilized methacrylated collagen solution; cm^2^, square centimeter; DMEM, Dubelcco’s modified Eagles medium; ECMs, extracellular matrix; EPCs, endothelial progenitor cells; FBS, fetal bovine serum; FDPCs, follicle dermal papillary cells; G, gauge; GelMA, gelatin-methacrylamide; GFP, green fluorescent protein; GSL-B_4_, griffonia gimplicifolia gectin I isolectin B_4_; h, hours; HA, hyaluronic acid; hASCs, human adipose tissue-derived stem cells; hDMECs, human dermal microvascular endothelial cells; hECs, human endothelial cells; hFBs, primary human fibroblasts; hKCs, human keratinocytes; hMCs, human melanocytes; hMSCs, human mesenchymal stem cells; hNKCs, human neonatal keratinocytes; hNFBs, human neonatal fibroblasts; hPCs, human pericytes; HUVECs, human umbilical vein endothelial cell; H&E: hematoxylin and eosin; ICAM, intercellular adhesion molecules; IU, international units; I2959, Irgacure D-2959 (2-hydroxy-1-[4-(2-hydroxyethoxy) phenyl]-2-methyl-1-propanone); kDa, kilodalton; kPa, kilopascal; m, meter; mFBs, mouse fibroblasts; min, minute; mg, milligram; mL, milliliter; mm, millimeter; mM, millimolar; MPa, megapascal; MW, molecular weight; mW, milliwatts; NaCl, sodium chloride; s, seconds; RFP, red fluorescent protein; RT, room temperature; SMA, smooth muscle actin; s-dECM, skin-derived decellularized extracellular matrix; SEM: scanning electron microscope; TG, transglutaminase; U, units; UV, ultraviolet; VCAM, Vascular cell adhesion protein; W, watts; *w*/*v*, weight/volume; µm, micrometer.

**Table 2:** Studies focused on printable biomaterials that expedite the manufacturing process.

**Alginate**						
**Reference**	**Biomaterial**	**Cell Phenotypes (Source, Density)**	**Rheology**	**Bioprinting Conditions**	**Post-Printing Processing**	**Bioprinted Construct Application/Evaluation**
Crook J.M. 2020 [33]	Alginate (5% *w*/*v*), Carboxymethyl chitosan (5% *w*/*v*), Agarose (1.5% *w*/*v*)	iPSCs (20–40 × 10^6^ cells)	NO	Needle Ø: 19 G, 1 mL syringe, Pressure: 0.3 bar, Speed: 9 mm/s, Temp: 15 °C	CaCl_2_ (2% *w*/*v*, 10 min, RT)	Immunophenotyping (OCT4, SSEA4, TRA-1-‘60, TRA-1-81), Cell viability
Motealleh A. 2019 [34]	Alginate and Nanocomposites (DXPPMO-L-Asp-Alg and DXPPMO-D-Asp-Alg)	hDFs and mFBs (10,000 cells)	NO	Not reported	CaCl_2_ (22.5 M, 10 min)	3D bioprinted triphasic chiral nanocomposite hydrogels to study the effect of the addition of nanocomposites and the chirality of enantiomers in cell activities. Cell morphology, adhesion, and migration
Ooi H.W. 2018 [35]	Alginate (2%), 5-Norbornene-2-methylamine and RGD Peptide Sequence (CGGGRGDS); photoinitiator and PEG linker	MFBs, ATDC5 Chondrocytes	YES	Metal needle Ø: 25 G Speed: 10 mm/s, Pressure: 30 kPa	UV (365 nm, 10 mW/cm^2^, 60 s)	Development of bioink with modified alginate, allowing its printability with low alginate concentration and high cell viability
Raddatz L. 2018 [36]	Alginate (0.5, 1, 2, 3 and 4% *w*/*v*)	hASCs and mFBs-GFP (5 × 10^6^ cells/mL)	YES	Nozzle Ø: 0.256 mm Temp. platform: 37 °C Temp. syringes: RT, Pressure: 90.3 mPa	CaCl_2_ (500 mM, nebulized)	Development of a calcium chloride nebulizer to reduce the negative impact of high concentrations of CaCl_2_ on cell-laden bioinks
Shi P. 2017 [37]	Alginate (2%, 5%, and 10%)	mFBs (5 × 10^6^ cells/mL)	YES	Nozzle Ø: 27 G	CaCl_2_ (100 mM, 5 min)	Analysis of the effect of hydrogel stiffness on cell activities of fibroblast in bioprinted cell-laden alginate hydrogels
Dubbin K. 2016 [38]	Alginate (2%), P1 peptide (2 mg) and C7 protein polymer (10%)	mFBs and hASCs (10 × 10^6^ cells/mL)	YES	Blunt-tipped nozzle Ø: 32 G, Pressure: 10 psi, Speed: 4 mm/s	CaCl_2_ (10 mM, 10 min)	Study of the effect of two crosslinking processes in two component bioink to ensure high cell viability
**Gelatin**						
**Reference**	**Biomaterial**	**Cell Phenotypes (Source, Density)**	**Rheology**	**Bioprinting Conditions**	**Post-Printing Processing**	**Bioprinted Construct Application/Evaluation**
Tigner T.J. 2020 [39]	GelNB (10% *w*/*v*), GelMA (10% *w*/*v*), LAP or I2959 (4.46 mM)	mFBs (2 × 10^6^ cells/mL)	YES	Needle Ø: 18 G; 5, 10 and 15 mm/s printing speed; 2–4 mm/s extrusion speed	Continuous exposure to UV (365 nm), Intensity: 5 mW/cm^2^ (LAP); 20 mW/cm^2^ (I2959)	Comparative analysis of photocrosslinkable gelatin derivatives (GelNB vs. GelMA) combined with different photoinitiators (LAP vs. I2959)
Pepelanova I. 2018 [40]	GelMA (5% *w*/*v*) and AlgHEMA (0, 1, 3% *w*/*v*) or SiNPs (0, 1, 2% *w*/*v*)	HASCs (1.5 × 10^6^ cells/mL)	YES	Needle Ø: 0.40 mm, Pressure: 2.8–3.8 psi, Temp: 30 °C or 37 °C, Speed: 260 mm/min	UV (365 nm, 1.2 J/cm^2^, 25 °C)	Improvement of extrusion bioprinting by adding biocompatibles additives to increase the hydrogel viscosity (SiNPs and the novel AlgHEMA). Hydrogel brings a cell-promoting microenvironment for hADSCs
Liu W. 2017 [41]	GelMA (3%, 4%, 5%) and Photoinitiator (0.5%)	HUVECs (4 × 10^6^ cells/mL)	YES	Cone-shaped nozzles and straight nozzle, Ø: 27 G; Temp: 21 °C; Speed: 400 mm/min 100 µL/min feeding rate	UV (3.95 W/cm^2^, 30 s)	Development of GelMA constructs that support cell viability, survival, and spreading
Ouyang L. 2017 [42]	MeHA (2.5 wt %), NorHA (2 wt %), GelMA (5 wt %), PEGDA (5 wt %), I2959 or LAP photoinitiator (0.05 wt %)	mFBs (2.5 × 10^6^ cells/mL)	NO	Coaxial system: Core needle Ø: 23/24 G; shell needle Ø: 18 G; Flow rate: 0.4 mL/h	In-situ crosslinking UV (10–15 mW/cm^2^) or visible light	Development of a extrusion technology to print simple or complex filaments (core/shell) using a general strategy for photocrosslinkable hydrogels
Rutz A.L. 2015 [43]	Gelatin type A (5% *w*/*v*), Fibrinogen (3% *w*/*v*), TGFß (5% *w*/*v*), 4-arm PEG amine (20% *w*/*v*) and GelMA (10% *w*/*v*)	HDFs, HUVECs, hMSCs	YES	Nozzle Ø: 200 μm Pressure: 1–2.5 bar Speed: 5 mm/s (1–2 h of bioink incubation prior to printing at 37 °C)	(I) UV (365 nm, 15–20 mW/cm^2^, 10 min); (II) Thrombin (10 U/mL) and CaCl_2_ (40 mM) for 30 min	Development of versatile and cell-compatible bioink printing method for creating soft, printable gels from a variety of synthetic and natural polymers
**Alginate + Gelatin**						
**Reference**	**Biomaterial**	**Cell phenotypes (Source, Density)**	**Rheology**	**Bioprinting Conditions**	**Post-Printing Processing**	**Bioprinted Construct Application/Evaluation**
Bociaga D. 2019 [44]	Alginate (5% *w*/*v*) gelatin (3–4% *w*/*v*)	hECs	YES	Flat-tip needle Ø: 430 µm, length: 16 mm) Temp: 34 °C, 37 °C, 40 °C. Thickness: 0.35 mm	CaCl_2_ (2%)	Control of mechanical properties, cell survival after extrusion, and degradation rate of hydrogels prepared in water vs. [DMEM + 10% FBS]
Compaan A.M. 2019 [45]	(I) Gelatin (5–10% *w*/*v*) and Alginate (2% *w*/*v*); (II) Gellan (0.5%), Gelatin (4%) and CaCl_2_·2H_2_O (0.1% *w*/*v*) (various gellan fluid bath formulations)	mFBs (5 × 10^6^ cells/mL)	YES	Gellan bath enabled extrusion bioprinting; Stainless steel tips Ø: 23 G, Speed: 2.5–10 mm/s; Thickness: 0.1–0.15 mm	*Enzyme-mediated covalent crosslinking*: TG (37 °C, 45 min); *Alginate structures*: CaCl_2_, 2 h *PEGDA structures:* UV, 15 min	Analysis of the versatility and advantages of using gellan gum-based fluid gel formulations as a support bath material for the bioprinting of 3D hydrogels and the addition of TG for the gelation of native gelatin. Analysis of postprinting stability with different crosslinking protocols. Living fibroblasts spread and multiply, cell extension and cell–cell contacts better with bioink II (Gellan)
Liu P. 2019 [46]	Alginate (2 wt %), Gelatin (15 wt %)	hAECs, WJMSCs 1 × 10^6^ cells/mL	YES	Pressure: 0.2 Mpa, Nozzle Ø: 0.33 μm, Speed: 7 mm/s, Temp: 30 °C	*During printing:* Instantaneous gelation at 4 °C; *After printing:* CaCl_2_ bath (2 wt %, 30 min, RT)	Cell phenotypes, gene expression microarrays: differentially expressed genes hAECs vs. hWJMSCs. Human AECs superior epithelial cells phenotype, WJMSCs superior angiogenic potential and fibroblastic phenotype. Uniform cell distribution. Cell viability > 95%
Giuseppe M.D. 2018 [47]	Performance of different alginate/gelatin blends, i.e., 9% Alg/6% Gel; 5% Alg/10% Gel; 7% Alg/8% Gel	sMSCs	YES	Nozzle Ø: 27 G, Speed: 5 mm/s, Temp: 25 °C	CaCl_2_ (300 mM, 15 min)	Optimized printability with alginate (7%)/gelatin (8%) (POI determination). Compressive modulus. Cell survival 92%
Li Z. 2018 [48]	Alginate (2.4%) Gelatin (12%) with varying solvent strengths	mESCs (1 × 10^7^ cells)	YES	Pre-cooling of the bioink at 0 °C for 30 min, Printing temp: 10 °C	CaCl_2_ (10%, 0 °C, 10 min)	Description of the effect of solvents on printability, mechanical properties, and cell behavior (viability, proliferation, aggregation, differentiation). Bioink designed for regenerating sweat glands
Liu W. 2018 [49]	*Sheath:* Alginate (1%), *Core*: GelMA, photoinitiator (0.2%) and CaCl_2_ (1%)	HUVECs, MCF-7, mFBs	YES	Coaxial system. 23 G core Ø: 23 G, Ø sheath: 28 G. Speed: 500 mm/min	UV (3.95 W/cm^2^)	Development of cell-laden constructs at low concentrations of GelMa (< 2%) Mechanical properties. Cell survival and proliferation
He Y. 2016 [50]	Alginate (2.5%) and Gelatin (8%)	L929 mFBs (1 × 10^6^ cells/mL)	YES	Temp: 37 °C nozzle and 5 °C substrate. Pressure: 20 KPa, nozzle Ø: 0.3 mm. Speed: 4.45 mm/s	CaCl_2_ (2% *w*/*v*, 5 min)	Identification of the most important parameters for good printability: viscosity range, air pressure, nozzle Ø, distance between nozzle and substrate. Control of printing quality. Diffusion within and between layers. Cell viability
Ouyang L. 2016 [51]	Gelatin (7.5% *w*/*v*) and Alginate (1% *w*/*v*)	mESCs	YES	Stainless steel needle Ø: 25 G, Extrusion flux: 0.68 uL/s. Temp: nozzle at 30 °C, chamber at 22.5 °C	CaCl_2_ (100 mM, 3 min)	Assessment of printability of gelatin/alginate bioinks. Shear stress determination. ESC viability: 95%, cell spreading
Wu Z. 2016 [52]	Alginate (1%), Gelatin (10%) and Collagen (from bovine Achilles tendon, 0.82 mg/mL)	hCECs (1 × 10^6^ cells/mL)	NO	Not reported	CaCl_2_ (3%, 37 °C, 3 min)	Incorporation of collagen to the bioink to precisely mimic tissue ECM yielding high cell viability and good printability. Effect of sodium citrate on degradation. Cell viability
**Others**						
**Reference**	**Biomaterial**	**Cell Phenotypes (Source, Density)**	**Rheology**	**Bioprinting Conditions**	**Post-Printing Processing**	**Bioprinted Construct Application/Evaluation**
**Cellulose**						
Montheil T. 2020 [53]	HPMC	hMSCs (1 × 10^6^ cells/mL)	YES	Pressure: 45 ± 5 psi; Conical tip Ø: 27 G, Temp: 37 °C, Speed: 10 mm/s	24 h, 37 °C	Determination of the printing window, Physicochemical analyses
Zidaric T. 2020 [54]	Alginate (3 wt %), CMC (3 wt %) and NFC (1.5 wt %)	hDFs (10^6^ cells/mL)	YES	Nozzle Ø: 0.25 mm	CaCl_2_ (pouring 2 wt % for 1 min)	Wettability, Swelling ratio, In vitro degradation, Cell viability
Mendes B.B. 2019 [55]	Aldehyde-CNC (2.88 wt %) and platelet lysate (160 mg/mL of total dry mass)	hASCs(2 × 10^6^/mL PL)	YES	Dual-extrusor with a static mixer, Stainless steel needle Ø: 27 G, Speed: 5 mm/s, Temp: 20 °C	h-thrombin from plasma (5 U/mL) CaCl_2_ (10 mM, 1 h, 37 °C)	Free-form fabrication, Hierarchical fibrillary architecture, Molecular diffusion, Cell viability > 90%, Metabolic activity, Collagen synthesis after 9 days
Law N. 2018 [56]	Hyaluronic acid-7 (0.25–2 wt %) and Methylcellulose (0.5–9 wt %)	sMSCs	YES	Pressure: 160–175 kpa, Ø: 23 G, Speed: 3 mm/s speed, Temp: extruder at 4 °C, plate at 37 °C	37 °C, 5% CO_2_, 1 h	Swelling and stability, Compression behavior, Cell viability post-printing, Long-term cell viability (2 weeks)
Li H. 2017 [57]	Alginate (3%), methylcellulose (9%) and CaCl_2_, Trisodium citrate to enhance interfacial adhesion	L929 mFBs (3 × 10^6^ cells/mL) in 15 mg/mL trisodium citrate	YES	Syringe 1: nozzle Ø: 25 G, Pressure: 4 bar; Syringe 2: nozzle Ø: 27 G, Pressure < 0.1 bar; Speed: 7.6–156.7 mm/s; Temp: 20 °C	CaCl_2_ bath (40 mg/mL, 10 min)	Printability, Mechanical properties, Degradation behavior, Thixotropic properties, Morphology, Cell viability > 95%
**Chitosan**						
Pisani S. 2020 [58]	Chitosan (4.5–6% *w*/*v*) and Gamma-PGA (2% *w*/*v*)	hDFs (2 × 10^5^ cell/mL)	YES	Needle Ø: 22 G and Ø: 25 G. Pressure (chitosan): 25–40 kPa and 5–10 kPa (Gamma-PGA). Speed: 600 mm/min Temp: 37 °C	No	Morphology, Stability (up to 35 d), Physicochemical characterization, Cell viability > 60%
Li Y. 2018 [59]	Hydroxypropil chitin (HPCH, 5 wt %, 0.4–0.6 mL) and Matrigel (0–0.3 mL)	hiPSCs (1 × 10^6^ cells/mL)	YES	Nozzle Ø: 260 μm (160–360 μm), Speed: 2–6 mm/s, Temp: 15 °C–37 °C	CaCl_2_ (1% *w*/*v*, 37 °C, 3 min)	Thermal sensitive hydrogel printability, Cell viability (day 0), Proliferation (day 7), Morphology (0–7 d), Aggregation (10 d), Apoptosis (day 1), Pluripotency (qRT-PCR, day 10)
**Hyaluronic Acid**						
Wang L.L. 2018 [60]	Nor-HA, HA-HYD, HA-ALD, I2959 photoinitiator (0.05%) and PETMA crosslinker	mFBs (2 × 10^6^ cells/mL)	YES	Nozzle Ø: 25 G, Speed: 40 mm/s	UV irradiation (365 nm, 10 mW/cm^2^, 2 min).	HA-HYD and HA-ALD characterization, Mechanical properties, Cell viability > 80%
**Pectin**						
Pereira R.F. 2018 [61]	PECMA (macromere conc. 1.5 or 2.5 wt %), I2959 (0.05 wt %), CaCl_2_ (0–5 mM)	hDFs	YES	Metal cylindrical nozzle Ø: 23 G; Temp: 20 °C; Construct, 15 layers	Dual crosslinking: UV photopolimerization (160 s, 7 mW/cm^2^), Ionic gelation (CaCl_2_, 5 mM 1 h under agitation)	Biofunctionalization of PECMA, Mechanical properties, Swelling, Cell viability and spreading, Deposition of ECM (fibronectin)
**Polyethylene Glycol**						
Rutz A.L. 2019 [62]	PEG-SH/PEG-NH2 inks (base polymer (20%) + PEG crosslinker (10%))	hDFs (2 × 10^6^ cells/mL)	YES	Stainless steel nozzle Ø: 200 µm, 2 mm length. Pressure: 5 bar	Covalent amine-activated ester crosslinking	Optimization of PEG bioinks, Mechanical properties, Cell viability
Xin S. 2019 [63]	PEG microgel produced by electrospraying and thiol-ene click chemistry	hMSCs (5 × 10^6^ cells/mL)	YES	Nozzles Ø: 840 and 600 µm	UV (60 mW/cm^2^, 365 nm, 3 min)	Gel morphology, Printability of complex structures, Cell viability up to 10 d

AlgHEMA, alginate derivatives; Asp, L(D)-Aspartic acid; cCNCs, carboxylated-cellulose nanocrystals; CMA, acid-solubilized methacrylated collagen solution; cm2, square centimeter; a-CNC, aldehyde- cellulose nanocrystals; CNC, cellulose nanocrystals; d, days; DXP, N,N’-bis(2,6-dimethylphenyl)perylene-3,4,9,10-tetracarboxylicdiimide; ECMs, extracellular matrix; FBS, fetal bovine serum; G, gauge; Gamma-PGA, gamma-poly(glutamic acid); GelMA, gelatin-methacrylamide; GelNB, gelatin-norbornene; GFP, green fluorescent protein; h, hours; HA, hyaluronic acid; HA-HYD, hyaluronic acid with hydrazides; HA-ALD, hyaluronic acid with aldehydes; hAECs, human amniotic epithelial cells; HAHYD, HA with hydrazone bonds; HAMC, hyaluronic acid and methylcellulose; hASCs, human adipose tissue-derived stem cells; hCECs, human corneal epithelial cells; hDFs, human dermal fibroblasts; hECs, human endothelial cells; HEPES, 4-(2-hydroxyethyl)-1-piperazineethanesulfonic acid; hESCs, human embryonic stem cells; hFBs, primary human fibroblasts; hMSCs, human mesenchymal stem cells; HPCH, hydroxypropyl chitin; HPMC, hybrid hydroxypropyl methyl cellulose; hiPSCs, human-induced pluripotent stem cell; HUVECs, human umbilical vein endothelial cell; H&E: Hematoxylin and eosin; IU, international units; I2959, Irgacure D-2959 (2-hydroxy-1-[4-(2-hydroxyethoxy) phenyl]-2-methyl-1-propanone); kDa, kilodalton; kPa, kilopascal; LAP, lithium phenyl-2,4,6-trimethylbenzoylphosphinate; m, meter; MCC, microcrystalline cellulose; MCF-7, Michigan Cancer Foundation-7 breast cancer cell line; MeHA, methacrylated hyaluronic acid; mESCs, mouse embryonic stem cells; mFBs, mouse fibroblasts; min, minute; mg, miligram; mL, milliliter; mm, millimeter; mM, millimolar; MNCs, mononuclear cells; MPa, megapascal; MW, molecular weight; mW, milliwatts; Na-Alg, sodium alginate; NaCl, sodium chloride; NaF, sodium fluoride; NC, chiral nanocomposite; -NH2, amine crosslinking; NorHA, functionalyzed HA with norbornene groups; PCL, polycaprolactone; PECMA, pectin methacrylate; PEG, polyethylene glycol; PEGDA, poly-(ethylene glycol) diacrylate; PEGX, PEG-crosslinker; PETMA, pentaerythritol tetramercaptoacetate; PMO, periodic mesoporous organosilica; PGA, polyglycolic acid; PBS, phosphate-buffered saline; s, seconds; RGD, tripeptide arginine–glycine–aspartate; RT, room temperature; SEM: scanning electron microscope; s, seconds; sMSCs, sheep mesenchymal stem cells; -SH, –thiol crosslinking; SiNPs, silicate nanoparticles; TEER, transepidermal electrical resistance; TG, transglutaminase; TGFß, transforming growth factor beta; TSC, trisodium citrate; U, units; UV, ultraviolet; WJMSCs, Wharton’s jelly-derived mesenchymal stem cells; W, watts; *w*/*v*, weight/volume; μm, micrometer.

**Table 3:** Technology readiness level (TRL) definition and bioprinting TRL adaptation. The definition of TRLs proposed in the EU framework program projects (Horizon 2020, H2020) are listed in column 2. The bioprinting TRLs, shown in column 3, were adapted from the Medical Device Scale [89,90]. 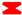 Indicates how far has evolved skin extrusion bioprinting; ●/○ indicates research category implementation/ non-implementation. The different colors group together, in more general terms, the different TRLs.

	TRL	Technology Readiness Level Scale Proposed by H2020	Bioprinting TRL Adaptation	Research		
				In Vitro	In Vivo Animal	In Vivo Human
**Basic research**	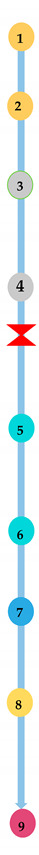	Basic principles observed	Review of the scientific literature to establish the starting point for the characterization of the new technology and procedure.	**○**	**○**	**○**
		Technology concept formulated	Development of hypothesis and experimental designs for addressing the related scientific issues.	**○**	**○**	**○**
**Applied research**		Experimental proof of concept	Beginning of the research. Identification of candidate and/or target. In vitro demonstration of activity of skin constructs. Generation of preliminary in vivo proof-of-concept efficacy data (non-GLP (Good Laboratory Practice)).	●	●	**○**
		Technology validated in lab	Optimization and Non-GLP in vivo demonstration of activity, toxicity and efficacy of the skin construct. Manufacture of the product at laboratory-scale (i.e., non-GMP (Good Manufacturing Practice))	●	●	**○**
**Product demonstration**		Technology validated in relevant environment	Advanced characterization of skin constructs (non-GLP in vivo studies, animal model, and assay development) Establishment of preliminary target product profiles. Initiation of the development of a scalable and reproducible manufacturing process according to GMP standards.	●	●	**○**
		Technology demonstrated in relevant environment	GMP Pilot Lot Production. Phase 1 clinical trial(s): Determination of an initial safety pharmacokinetics and immunogenicity as well as other properties of the clinical product.	●	●	●
		System prototype demonstration in operational environment	Scale-up and initiation of GMP process validation. Phase 2 clinical trial(s).	●	●	●
		System complete and qualified	Completion of GMP validation and consistency lot manufacturing. Animal efficacy studies, Phase 3 clinical trials, as well as any other extended clinical safety trials that are appropriate for the product Regulatory issues or product licensure.	**○**	●	●
**Competitive manufacturing**		Actual system proven in operational environment	Phase 4 studies (post-marketing commitments), safety surveillance, studies to support use in special populations, and clinical trials to confirm safety and efficacy as feasible and appropriate. Maintenance of manufacturing capability.	**○**	**○**	**○**
